# Expression Profile of p53 and p21 in Large Bowel Mucosa as Biomarkers of Inflammatory-Related Carcinogenesis in Ulcerative Colitis

**DOI:** 10.1155/2016/3625279

**Published:** 2016-08-07

**Authors:** Cristiana Popp, Luciana Nichita, Theodor Voiosu, Alexandra Bastian, Mirela Cioplea, Gianina Micu, Gabriel Pop, Liana Sticlaru, Andreea Bengus, Andrei Voiosu, Radu Bogdan Mateescu

**Affiliations:** ^1^Colentina University Hospital, 020125 Bucharest, Romania; ^2^Carol Davila University of Medicine and Pharmacy, Bucharest, Romania

## Abstract

Ulcerative colitis (UC) is a chronic, relapsing inflammatory bowel disease that slightly increases the risk of colorectal cancer in patients with long-standing extended disease. Overexpression of p53 and p21 in colonic epithelia is usually detected in UC patients when no dysplasia is histologically seen and it is used by pathologists as a discriminator between regenerative changes and intraepithelial neoplasia, as well as a tissue biomarker useful to predict the risk of evolution toward malignancy. We present a one-year prospective observational study including a cohort of 45 patients with UC; p53 and p21 were evaluated in epithelial cells. p53 was positive in 74 samples revealed in 5% to 90% of epithelial cells, while 63 biopsies had strong positivity for p21 in 5% to 50% of epithelial cells. Architectural distortion was significantly correlated with p53 overexpression in epithelial cells. Thus, we consider that architectural distortion is a good substitute for p53 and p21 expression. We recommend use of p53 as the most valuable tissue biomarker in surveillance of UC patients, identifying the patients with higher risk for dysplasia. Association of p21 is also recommended for a better quantification of risk and for diminishing the false-negative results.

## 1. Introduction

Ulcerative colitis (UC) is a chronic, relapsing inflammatory bowel disease with increasing incidence and prevalence in Europe. Epidemiological studies are indicating that multiple characteristics of western way of life (high hygiene standard, overexposure to pollutants, and stress) are at least risk factors for this disease, suggesting that the number of cases will constantly increase in the next decades [1–3]. Also, the fact that UC has a low rate of mortality, but also is incurable for now, induces an ascending rate of prevalence for the disease.

Multiple studies are indicating that UC slightly increases the risk of colorectal cancer in patients with long-standing extended disease. Accepted risk of colorectal cancer is about 4/1000 per person year duration [4]. Also, the mortality of colorectal carcinoma in patients with UC is higher than that for sporadic cases [5–8]. These data, along with the fact that there is an increasing number of older patients, with long-standing disease, history of multiple flares, and increased risk for epithelial malignancy, are emphasizing the importance of proper surveillance of UC patients in order to prevent and/or early-diagnose intraepithelial and invasive neoplasia. Although there are insufficient data to sustain the importance of colonoscopic surveillance in preventing carcinoma in UC, cohort studies have demonstrated reduced risk of malignancy and improved survival in patients with UC undergoing routine colonoscopy at 2 to 3 years intervals, beginning 8 years after diagnosis [9]. Since intraepithelial neoplasia is difficult to identify with usual colonoscopic techniques, the diagnosis requires advanced endoscopic procedures or multiple biopsies (minimum 36 each time) [10]. Both alternatives are increasing the costs of this surveillance and have a significant risk of false-negative results. For this reason, studies of expression proteomics are needed to validate some tissue biomarkers that can be used to evaluate progression toward malignancy in patients with UC.

Cause of carcinogenesis in UC is considered chronic inflammation of colonic mucosa with increased cell turnover and accelerated reepithelialization that leads, in the end, to a higher risk of errors in the cell cycle repair. UC flairs are characterized by a predominant neutrophilic infiltration with crypt abscesses and ulceration of the epithelium, on the background of chronic inflammation. Between flairs, usually there is a state of inactive mucosal inflammation characterized by predominance of lymphocytes. Over time, epithelial colonic cells suffer from genomic instability induced by oxidative stress, linked to chronic inflammation. Inflammatory infiltrate in UC generates oxygen radicals and nitrogen species that affect numerous metabolic processes involved in cell repair [11, 12].

Most important mutation occurs early in UC and involves p53 gene. Overexpression of p53 in colonic epithelia is usually detected in UC patients when no dysplasia is histologically seen and it is used by pathologists as a discriminator between regenerative changes and intraepithelial neoplasia, as well as a tissue biomarker useful to predict the risk of evolution toward malignancy. A high frequency of p53 mutations has been found in chronic UC patients with severe disease who were not diagnosed with cancer [13–15].

p21 oncoprotein expression is also persistently increased in epithelial cells in UC, in active phase, and also in remission, especially when it is associated with cryptic atrophy. This feature is considered to be the result of mutation in* ras* gene that plays an important role in UC-related carcinogenesis. Other studies linked p21 upregulation to p53 mutation, finding that is more frequent in UC-related carcinomas than in sporadic cases [16–18].

Colorectal carcinoma arising on the ground of UC is, commonly, the result of a multistep process including inflamed mucosa without dysplasia, then low-grade dysplasia, high-grade dysplasia, and finally invasive adenocarcinoma, although each of the premalignant lesions can evolve directly towards carcinoma, without the intermediate steps [19, 20].

UC-related carcinoma differs from sporadic one because, in most cases, preinvasive and invasive lesions are multifocal, small, and flat making detection more difficult. The promising role of expression proteomics is highlighted by the fact that genetic abnormalities such as alterations in the p53, bcl-2, and* K-ras* genes, and their tissue expression is still present [13, 21].

This study is evaluating the tissue expression of p53 and p21 in patients with UC, in order to identify the natural evolution of these biomarkers and their relationship with carcinogenesis. A proper understanding of the importance of these markers should allow a better stratification of UC patients according to their risk for dysplasia and invasive carcinoma, in order to personalize their treatment and surveillance.

## 2. Materials and Methods

This study is an observational prospective cohort study that included 45 consecutive patients with clinical, endoscopic, histologic, and imagistic diagnosis of UC in their first 6 years of the disease. Patients were enrolled for close clinical, biochemical, endoscopic, and histologic surveillance. All patients were treated according to national and international guidelines for the disease and pathologists were blinded to the therapeutical approach.

*Criteria of Exclusion*. Criteria of exclusion were as follows: malignancies (invasive or preinvasive) in the moment of enrolling and refusal of signing an informed consent.

All patients underwent a thorough medical history, complete clinical examination, and endoscopic examination (ileocolonoscopy with narrow band imaging and magnification chromoendoscopy with complete video and photo documentation). From each patient, multiple biopsies of normal and injured mucosa were taken, including samples of rectal mucosa and ileal mucosa, using EndoKit for proper orientation of mucosa. Usually from each patient 3 or 4 biopsies were performed in each visit (minimum of two biopsies from the large bowel, one from the most injured area and one from an area with normal appearance. In all cases one piece of rectum mucosa was harvested. Also, an ileal biopsy was taken in all cases). In 14 patients (5 in the first presentation and 9 in the second), additional biopsies were taken from areas suspected of dysplasia. All tissue fragments were immediately immersed in 10% buffered formalin and then routinely processed (24-hour fixation in 10% buffered formalin, one hour water rinse, dehydration in 3 baths of 80%, 90%, and 96% ethanol for 6 hours each, then two one-hour baths of absolute ethanol, then clearing with toluene for one hour at 58°C, and, finally, 3 one-hour baths of paraffin at 58°C). The samples were paraffin embedded and two pairs of 3 *μ*m sections from two levels were obtained for each biopsy. The slides were stained routinely with hematoxylin-eosin and then examined and a histopathological diagnosis was formulated. If necessary, additional special stains were performed: periodic acid Schiff stain (PAS), Giemsa stain, Masson's trichrome stain, and Ziehl-Neelsen.

All of these procedures were repeated after a year (12 months) of follow-up for each patient included in our study.

These activities were carried in perfect compliance with national and European research laws and professional deontology and were approved by Colentina University Hospital (CUH) ethical committee.

After histological diagnosis, all tissue samples from the first and from the second visit were prepared for being evaluated, in research settings, as follows.

First, manual tissue multiarray blocks were created. For each patient, the most significant tissue sample from each of the two visits (a sample with the most severe inflammation was used, including at least 10 crypts) was extracted from the paraffin block and then reembedded into a single recipient block, each including 6 samples from 3 patients.

From each multitissue block several histological slides were sectioned for routine stain (hematoxylin-eosin), special stains (PAS, Giemsa, and Masson's trichrome stain), and immunohistochemical tests for p53 and p21.

For immunohistochemistry we used indirect tristadial method, using peroxidase and alkaline phosphatase as enzymes and DAB for peroxidase (brown)/AEC (red) and “fast red” and “fast blue” for alkaline phosphatase as substrates.

The sections were deparaffinized and hydrated on automatic stainer. Then, the following steps were made:

(1) Washing with TBS buffer, pH 7.4, 3 washes, 5 minutes each.
(2) Pretreatment: boiling in microwave oven in citrate buffer pH 8 or EDTA buffer pH 10, as specified for each antibody.
(3) Washing with TBS buffer, pH 7.4, 3 washes, 5 minutes each.
(4) Hydrogen Peroxide Block, LabVision, for 15 minutes.
(5) Washing with TBS buffer, pH 7.4, 3 washes, 5 minutes each.
(6) Blocking of nonspecific binding with Ultra V Block LabVision, 10 minutes.
(7) Washing with TBS buffer, pH 7.4, 3 washes, 5 minutes each.
(8) Primary antibody to each section for 1 hour in wet room.
(9) Washing with TBS buffer, pH 7.4, 3 washes, 5 minutes each.
(10) Secondary antibody biotinylate to each section 10 minutes (anti-mouse, Ultravision Detection Systems LabVision).
(11) Washing with TBS buffer, pH 7.4, 3 washes, 5 minutes each.
(12) Streptavidin Peroxidase LabVision, 10 minutes.
(13) Washing with TBS buffer, pH 7.4, 3 washes, 5 minutes each.
(14) Developing with DAB Plus Substrate System, LabVision.
(15) Washing with running water.
(16) Counterstain with hemalaum Meyer.
(17) Dehydration and drying.
(18) Mounting in Entellan.

Multiple histological and immunohistochemical parameters were evaluated and quantified by two independent fully trained pathologists, using preestablished scales.

**Figure 1:**
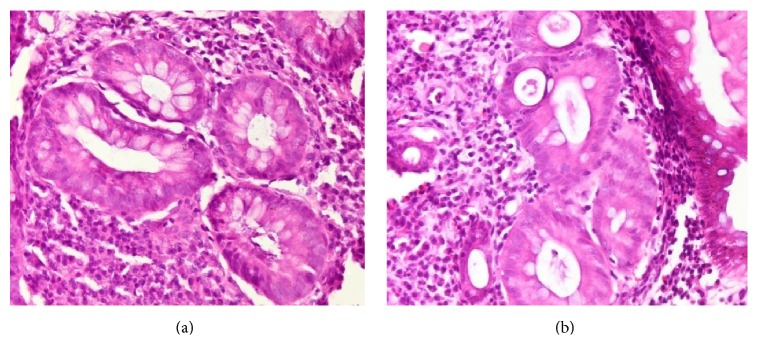
Regenerative epithelial changes (a) versus low-grade dysplasia (b) in UC. Note in (a) preservation of crypt architecture as well as nuclear polarization. In (b) crypt is distorted and epithelial cells have lost nuclear polarity.

Dysplasia was defined as “unequivocal neoplasia of the epithelium confined to the basement membrane, without invasion into the lamina propria” and was evaluated using the usual scale: negative for dysplasia, indefinite for dysplasia, low-grade dysplasia, high-grade dysplasia, and invasive carcinoma [22]. Consensus was reached in all cases by the initial two pathologists (Figure 1).

For p53 and p21 only intensely stained cells were quantified, results being expressed as percent from the epithelial cells examined on each section. If differences between the results of the two independent examinations were below 10 percentage points, the lowest value was taken. If the difference was higher, the slide was examined simultaneously with a third senior pathology and consensus was reached (lowest value that had complete agreement).

As control group we used 12 samples of normal colonic mucosa harvested from patients without inflammatory bowel disease (distant resection margins from sporadic colonic carcinomas). Each control sample underwent the same standardized procedures and was examined by the same two pathologists. Parameters identified were considered as normal counterparts and used as standard for evaluation of cohort samples.

*Study Limitations*. (a) The follow-up period (12 month) was somehow too short to assess the risk of development of cancer in these high-risk patients. Although, this study obtained some significant results correlating mucosal expression of p53 and p21 with other factors that indicate progression towards malignancy. (b) Patients included were in their first 6 years of the disease, when dysplasia is rare. But this study aimed to find subtle changes ratable before the occurrence of dysplasia, changes that can be used to identify patients with higher risk. (c) The studied group is pretty small, including only 45 patients, but this value is above the minimum needed for statistical significance, and all patients had a certain diagnosis of UC, and, also, all patients were submitted to a complex, multidisciplinary surveillance that offered significant data.

## 3. Results and Discussion

### 3.1. Data about the Cohort

The studied cohort included 45 patients, 31 men and 14 women. Only one patient had, on the second biopsy, an area of low-grade dysplasia, identified by chromoendoscopy and confirmed microscopically. No invasive carcinoma arose during this 12-month study.

Patients' evolution during the study was considered as follows:

- Favorable outcome: no relapse or complications during the study and a less severe clinical status (according Truelove & Witts classification) at the second presentation [23].
- Unfavorable outcome: all patients that did not fulfill the above criteria.

**Figure 2:**
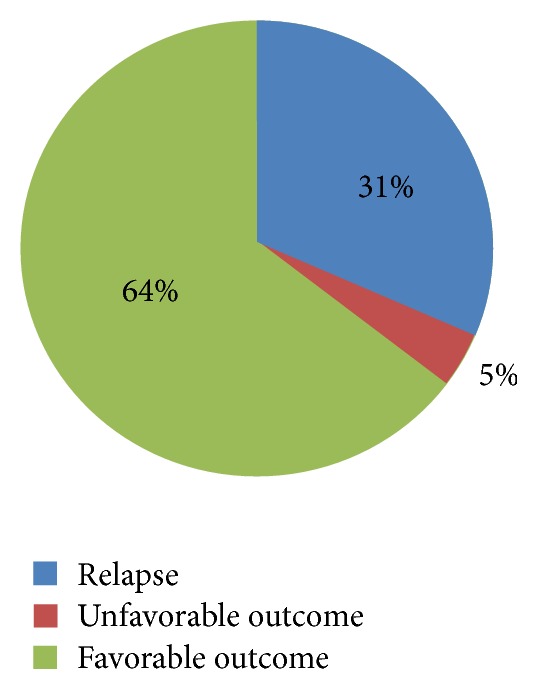
Patients' evolution during the study. Note that most of the patients had a favorable outcome.

Thus, 16 patients (~35%) had unfavorable outcome, while 29 patients (~65%) had favorable outcome (Figure 2). From the first group, 14 patients had a relapse (defined as recurrence of significant clinical and endoscopic activity of the disease after more than 6 months of remission), relapse being the most frequent negative event that interferes with patients' outcome in UC [24]. The other two patients did not acquire remission during the study and had a more severe clinical and endoscopic score in the second evaluation. From the 14 patients with relapse, two had additional complications: one tuberculous ileocolitis and one low-grade dysplasia of the rectum.

The risk for relapse in patients with UC is about 70% per year in the lack of treatment, but, in treated patients, this risk is between 23 and 33%, similar to our study [25].

### 3.2. Extension of the Disease

**Figure 3:**
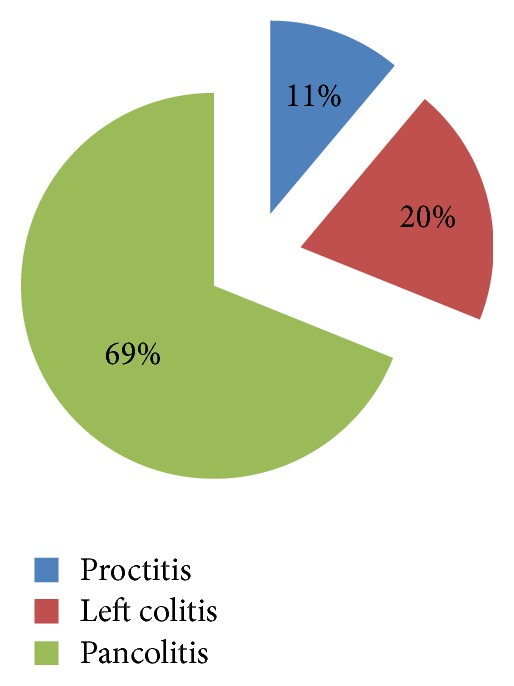
Disease extent at first presentation. Note that most patients had extensive disease, defined as pancolitis.

One of the most significant parameters that influence the risk of dysplasia is the extension of the disease. Patients with pancolitis had a more severe outcome and a higher risk of dysplasia, while proctitis is associated with a better prognosis and a reduced rate of morbidity and malignancies [4, 26]. In our cohort, 31 patients, representing 68.69%, had pancolitis, while only 5 patients (11.11%) had proctitis and 9 patients (20%) had left colitis (Figure 3).

**Figure 4:**
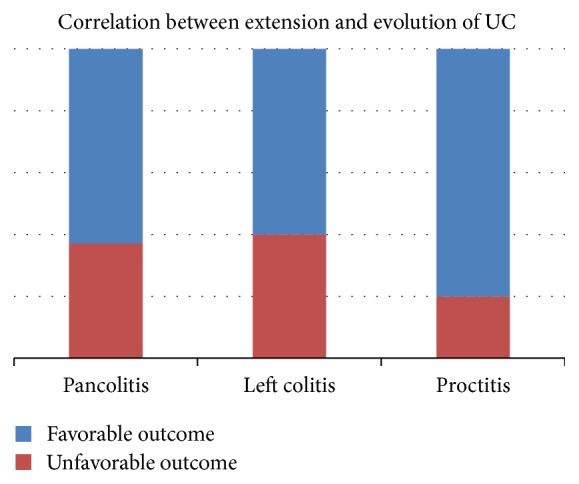
Correlation between patients' outcome and extension of the disease at first presentation.

As expected, 15 patients with extensive disease (left colitis and pancolitis) had unfavorable outcome, while only 2 patients with proctitis had the same evolution (Figure 4). This correlation was statistically significant (Fisher two-tailed test *p* = 0.036). This situation has multiple explanations, the most important being the availability of topical medication which are more efficient and have a lower rate of drug resistance [26, 27].

### 3.3. Concordance of the Most Severe Affected Area

**Figure 5:**
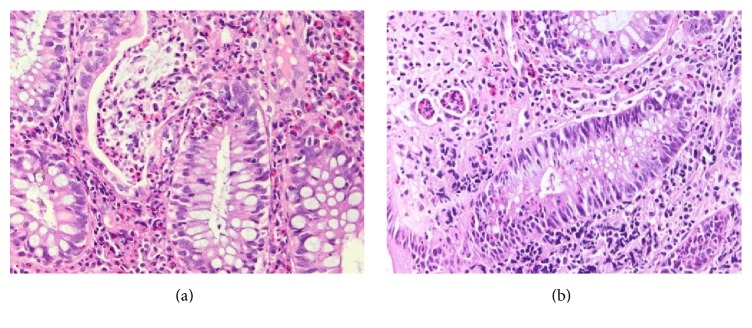
Severe inflammation in a patient with active ulcerative colitis. (a) Severe cryptitis with crypt destruction. (b) Cryptitis, crypt abscesses, and epithelial regenerative changes.

**Figure 6:**
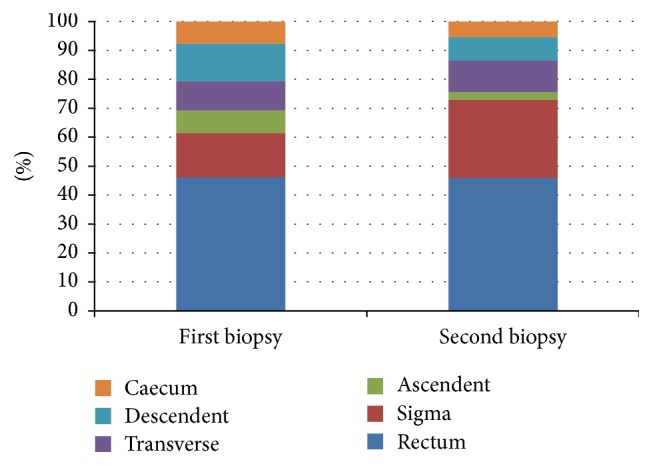
Presentation of the most affected area in the two evaluations. Rectum is always the most affected segment. Note a high concordance between the two biopsies, indicating the lesions are quite fixed in UC, regardless of patients' evolution.

Also, an important impact on the risk of dysplasia has the duration of severe inflammation. It is more probable that an area of the mucosa is exposed to intensive oxidative stress for a prolonged period to develop DNA damage with neoplastic changes. In our study, the concordance of the most severe affected area was as high as 78% (35 patients). Usually, in UC, rectum is the most affected segment, and our study confirmed this feature (Figure 5). From all patients, regardless of their evolution, 40 percent (18 patients) had the most inflamed area in both investigations in the rectum (Figure 6). In the late stages of the disease, rectum can be spared by the inflammation but remains the higher risk for dysplasia of local mucosa [28]. This high concordance indicates that in UC the same areas are usually affected, even in relapse after remission. These areas should be examined with additional attention in screening for dysplasia, since they are the most prone to having cellular mutations.

### 3.4. Evaluation of p53 and p21/Waf Expression

p53 and p21 are important proteins involved in normal settings, in avoidance of cellular mutations. p53 is a tumor suppressor, described as “the guardian of the genome,” regulating gene expression to prevent mutations of the genome and inducing apoptosis in case of DNA damage repair failure. It is the most frequently abnormal protein in human cancer [29]. p21 is a cyclin-dependent kinase inhibitor, inducing growth arrest of cells with DNA damage, usually controlled by protein p53. Both are important biomarkers used to confirm dysplasia lesions. They also have prognostic significance; patients with significant and prolonged overexpression of p53 and p21 have a higher risk of developing dysplasia [30].

**Figure 7:**
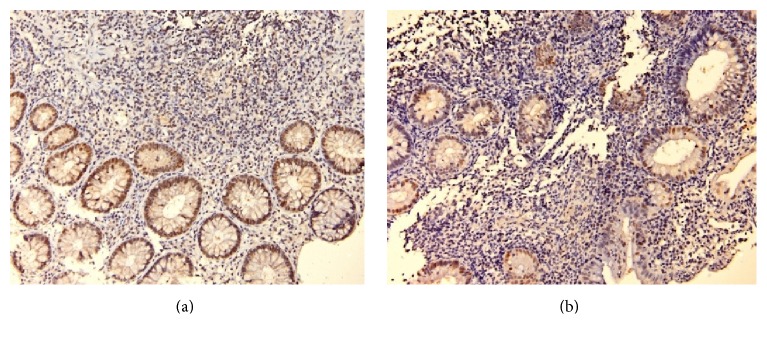
p53 (a) and p21 (b) positive in colonic epithelium in patients with UC.

In our study, we quantified the percent of epithelial cells with strong positivity for p53 and p21 (indicating mutations of these proteins). p53 was negative in 16 biopsies (9 from the first presentation and 7 from the second one). The rest of 74 samples revealed intense positivity for p53 in 5% to 90% of epithelial cells (maximal value was identified in the area of low-grade dysplasia) (Figure 7).

**Figure 8:**
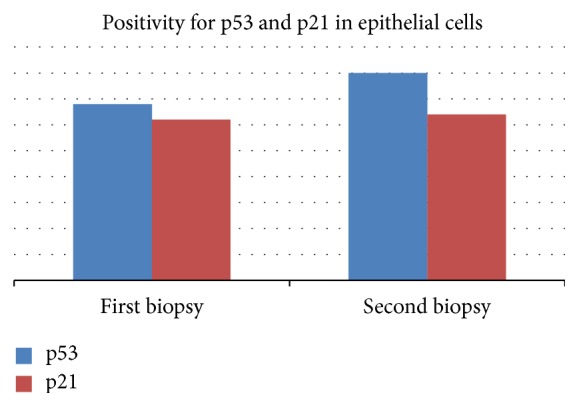
Evolution of p53 and p21 expression in epithelial cells in the 12 months of the study.

Meanwhile, 27 biopsies were negative for p21 (18 from the first presentation and 9 from the second one). In the rest of samples, 63, we had strong positivity for p21 in 5% to 50% of epithelial cells. Expression and evolution of p53 and p21 did not correlate significantly with patients' outcome or risk of relapse (Figure 8).

**Figure 9:**
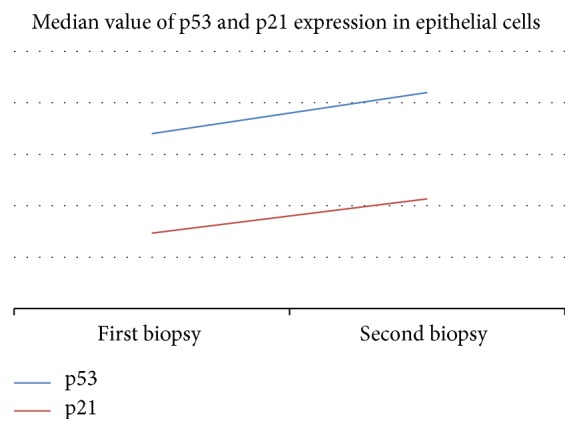
Evolution of median expression of p53 and p21.

Median percent of p53 positive cells was 17 for the first biopsy and 21 for the second one (no statistical significance). For p21, median percent of positive cells was 7.33 for the first biopsy and 10.67 for the second one (no statistical significance also) (Figure 9).

Correlated overexpression of p53 and p21 in epithelial cells in UC indicates accumulation of cellular mutation triggered by oxidative stress and build-up of toxic products in stromal microenvironment of colonic mucosa. Therefore, p53 and p21 are two members of expression proteomics family that respond to the need of pathologists and gastroenterologists to keep under control premalignant lesions of UC patients, identifying patients and areas with high risk of evolution towards malignancy, even before the apparition of unequivocal dysplasia.

Mitsuhashi et al. [31] identified a significant correlation between p53 and p21 expression and architectural distortion, concluding that DNA damages are accumulating in the same time with architectural abnormalities, both being results of oxidative on mucosal stroma and epithelium. We examined, also, the presence of this concordance. In our study, architectural distortion was significantly correlated with p53 overexpression in epithelial cells (two-tailed *t*-test *p* = 0.0251) and, also, with p21 overexpression (two-tailed *t*-test *p* = 0.0035). Thus, we consider that architectural distortion is a good substitute for p53 and p21 expression and can be used instead of immunohistochemical analysis in samples without dysplasia. However, significant worsening of architectural distortion, especially in patients with long-standing disease, compels a more careful endoscopic examination of mucosa in order to identify areas of dysplasia and evaluation of p53 and p21 status of mucosa [17, 32].

## 4. Conclusions

Ulcerative colitis has a high but preventable risk of evolution towards colonic adenocarcinoma; therefore, prevention and early diagnosis of dysplasia are mandatory. p53 and p21 are tissue biomarkers not only suitable for routine surveillance, but also for stratification of UC patients in risk categories with a more personalized approach.

Despite being rare, dysplasia is a severe event in UC evolution, frequently imposing prophylactic colectomy. It also has an unknown risk to escape surveillance and to evolve silently towards invasive carcinoma [33]. Our data recommend association of p53 with p21 to increase value of p53 as tissue biomarker used for identification of patients with higher risk for dysplasia. Association of p21 is also recommended for a better quantification of risk and for diminishing the false-negative results.
